# Partially replacing cyclophosphamide with bendamustine in combination with cyclosporine A improves survival and reduces xenogeneic graft-versus-host-disease

**DOI:** 10.3389/fimmu.2022.1045710

**Published:** 2023-01-09

**Authors:** Kristy E. Gilman, Megan J. Cracchiolo, Andrew P. Matiatos, Dan W. Davini, Richard J. Simpson, Emmanuel Katsanis

**Affiliations:** ^1^ Department of Pediatrics, University of Arizona, Tucson, AZ, United States; ^2^ Department of Immunobiology, University of Arizona, Tucson, AZ, United States; ^3^ School of Nutritional Sciences and Wellness, University of Arizona, Tucson, AZ, United States; ^4^ The University of Arizona Cancer Center, Tucson, AZ, United States; ^5^ Department of Medicine, University of Arizona, Tucson, AZ, United States; ^6^ Department of Pathology, University of Arizona, Tucson, AZ, United States

**Keywords:** allogeneic hematopoietic cell transplantation, bendamustine, cyclophosphamide, xenogeneic, graft-versus-host disease

## Abstract

**Introduction:**

The use of allogeneic hematopoietic cell transplantation (allo-HCT) for treating hematological disorders is increasing, but the development of graft-versus-host disease (GvHD) remains a major cause of morbidity and mortality. The use of post-transplant cyclophosphamide (CY) has significantly improved outcomes following allo-HCT, but complications of viral reactivation due to delayed immune reconstitution or relapse remain. Other laboratories are evaluating the potential benefit of lowering the dose of CY given post-transplant, whereas our laboratory has been focusing on whether partially replacing CY with another DNA alkylating agent, bendamustine (BEN) may be advantageous in improving outcomes with allo-HCT.

**Methods:**

Here, we utilized a xenogeneic GvHD (xGvHD) model in which immunodeficient NSG mice are infused with human peripheral blood mononuclear cells (PBMCs).

**Results:**

We show that a lower dose of CY (25 mg/kg) given on days +3 and +4 or CY (75 mg/kg) given on only day +3 post-PBMC infusion is not sufficient for improving survival from xGvHD, but can be improved with the addition of BEN (15 mg/kg) on day +4 to day +3 CY (75 mg/kg). CY/BEN treated mice when combined with cyclosporine A (CSA) (10mg/kg daily from days +5 to +18 and thrice weekly thereafter), had improved outcomes over CY/CY +CSA treated mice. Infiltration of GvHD target organs was reduced in both CY/CY and CY/BEN treatment groups versus those receiving no treatment. CY/CY +CSA mice exhibited more severe xGvHD at day 10, marked by decreased serum albumin and increased intestinal permeability. CY/BEN treated mice had reductions in naïve, effector memory and Th17 polarized T cells. RNAseq analysis of splenocytes isolated from CY/CY and CY/BEN treated animals revealed increased gene set enrichment in multiple KEGG pathways related to cell migration, proliferation/differentiation, and inflammatory pathways, among others with CY/BEN treatment.

**Conclusion:**

Together, we illustrate that the use of CY/BEN is safe and shows similar control of xGvHD to CY/CY, but when combined with CSA, survival with CY/BEN is significantly prolonged compared to CY/CY.

## Introduction

Allogeneic hematopoietic cell transplantation (allo-HCT) can be a curative treatment for hematological malignancies and other diseases, but the development of graft-versus-host disease (GvHD) can lead to morbidity and mortality. Finding ways to limit GvHD development while maintaining the graft-versus-tumor (GvT) effect in patients with hematological malignancies receiving allo-HCT is essential to improve patient outcomes.

Use of haploidentical-HCT (haplo-HCT), has rapidly increased in the last decade, largely due to the widespread use of post-transplant cyclophosphamide (PT-CY) which effectively eliminates alloreactive T cells (1, 2). However, this treatment often delays immune reconstitution, which can significantly increase the risk of viral reactivation or relapse due to suppressed GvT (3–7). While other laboratories are evaluating the potential benefits of a reduced dose of CY following haplo-HCT (8–11), we have been investigating replacing PT-CY with another DNA alkylating agent, bendamustine (BEN). We have shown that using BEN as either a component of a pre-transplant conditioning regimen or when used in a post-transplant setting led to equal or improved survival in mice undergoing major histocompatibility complex (MHC) mismatched or haploidentical bone marrow transplants (haplo-BMT), respectively (12, 13). BEN was shown to modulate immune cell number and function in transplanted mice, with increases in regulatory-like dendritic cells (DCs) (14, 15) and myeloid-derived suppressor cells (MDSCs) (16), reduced proliferation of B and T cells, and a higher presence of T cells that are tolerized towards host MHC molecules (12). Importantly, BEN treated animals, in both pre-transplant and post-transplant settings, had superior GvT effects with reduced tumor burden and improved survival compared to those treated with CY (12, 13). The combination of these data led to initiation of a first-in-human phase I clinical trial at our institution utilizing BEN for post-transplant GvHD prophylaxis (17, 18). Completion of the phase Ia 3 + 3 dose-escalation component of the trial indicated the optimal dosing of BEN was when given following a single day of PT-CY, with CY given on day +3 and BEN given on day +4 (CY/BEN) following haplo-BMT which is the basis for the dosing utilized in this study (19).

In the current study, we aimed to illustrate the safety and efficacy of CY/BEN utilizing a xenogeneic GvHD (xGvHD) model following infusion of human peripheral blood mononuclear cells (PBMCs) in immunodeficient mice. Since PT-CY is clinically combined with calcineurin inhibitors (CNIs), we therefore also evaluated the safety of combining these chemotherapy drugs with cyclosporine A (CSA). Herein, we demonstrate the efficacy of CY/BEN and CY/BEN +CSA in controlling xGvHD and improving survival, in a xenogeneic NSG model.

## Materials and methods

### Mice

NOD.Cg-Prkdc^scid^ Il2rg^tm1Wjl^/SzJ (NSG mice, Jackson Labs stock no. 005557, Bar Harbor, ME) were purchased and bred at the University of Arizona Experimental Mouse Shared Resource. Animals had *ad libitum* access to food and water and were maintained on a 12-hour light/dark cycle. Six-to-twelve-week-old female mice were used for experiments. Mice were randomized into groups by evenly distributing weights across treatment groups. All experimental plans were approved by the University of Arizona Institutional Animal Care and Use Committee (IACUC).

### Humanized BMT mouse model

NSG mice were irradiated with 150 cGy by a Cesium^137^ or RadSource X-ray irradiator one day prior to PBMC infusion. On day 0, mice received 5 x 10^6^ fresh ficoll-separated human PBMCs collected from a consented healthy donor, *via* tail vein injection. On days 3 and 4, indicated groups of mice received either intraperitoneal (IP) injections of cyclophosphamide (CY, Sigma, no. C0768, 25mg/kg, 75mg/kg, or 150mg/kg indicated in on graphs and in figure legends) solubilized in sterile water and diluted in 0.9% saline, or intravenous injections of bendamustine (BEN, SelleckChem no. S1212, 15mg/kg) solubilized in DMSO and diluted in PBS with 0.2% carboxymethylcellulose and 0.25% polysorbate 80. On days 5-18 and then thrice weekly after, mice received IP injections of cyclosporine A (CSA, SelleckChem no. S2286, 10mg/kg/day) solubilized in DMSO then diluted in 30% polyethylene glycol 300 (PEG300, SelleckChem no., S6704) and 5% polysorbate 80 in sterile water or vehicle (2% DMSO, 30% PEG300, 5% polysorbate 80 in sterile water). Mice were evaluated twice weekly for clinical signs of GvHD including activity, fur, posture, skin, and weight loss, measured on a scale of 0 to 2. Survival was monitored daily. Mice were removed from studies once reaching >30% weight loss at two consecutive weigh-ins or when moribund. Averages of weight or GvHD scores used the final score of deceased mice that was carried forward for the remainder of the study. Once greater than two thirds of the animals in a given group were deceased, the remaining mice were censored from graphs of averages of weight change or GvHD scores.

### Blood chemistry metabolic panel

Blood was collected into BD microtainer lithium-heparin tubes (BD, no. 365965) from mice in indicated groups and plasma was isolated following centrifugation at 10,000RPM for 10 minutes at room temperature. Then, a 12-item comprehensive chemistry panel (Heska, no. 054665) was run on a Dri-Chem 7000 Veterinary Chemistry Analyzer (Heska, Loveland, CO). Normal reference values for mice are listed on graphs in gray and indicated by gray shaded regions where applicable.

### Tissue harvest and preparation

Mice were bled *via* tail vein at indicated timepoints following PBMC infusion. Red blood cells (RBCs) were lysed for 15 minutes at room temperature in 1X lysis buffer (BD, no. BDB555899), quenched with media containing 5-10% fetal bovine serum (FBS, Millipore, no. TMS-013-B, lot VP1705185), washed and then resuspended in flow buffer (0.5% FBS in PBS) for subsequent flow cytometry staining. Alternatively, plasma samples were collected from animals by addition of either heparin or acid-citrate-dextrose solution, followed by centrifugation at 10,000 RPM for 10 minutes. Spleens were harvested at indicated timepoints, minced and manually disrupted to single-cell suspensions by filtering through a 100 μm strainer twice. Bone marrow was collected from hind limbs and run through a 100 μm strainer. RBCs from spleen and bone marrow were lysed for one minute in 1X lysis buffer, quenched with FBS containing media, resuspended in flow buffer and stained as described below for flow cytometry. At days 10 and 31, skin, liver and intestinal tissues were collected for histopathological and immunohistochemical analysis. Livers were rinsed of RBCs in 40mL ice-cold PBS. Skin sections were shaved and carefully excised from the middorsal region on the posterior side of the animal. Intestines were excised, flushed with ice-cold PBS and sections of duodenum, ileum, jejunum and colon were collected. Skin, liver and intestinal samples were fixed in 10% neutral buffered formalin overnight at 4°C and transferred to 70% ethanol the following day. Samples were then dehydrated, cleared, embedded in paraffin, and sectioned at 5 μm at the University of Arizona Tissue Acquisition, Cell and Molecular Analysis Shared Resource.

### Flow cytometry

Fc receptors of human (Biolegend, no. 422302) and mouse (eBioscience, no. 501129520) origin were blocked for 15 minutes, then cells were incubated in the following antibodies: anti-murine CD45 (mCD45, clone 30-F11, Biolegend, no. 103122), anti-human CD45 (hCD45, clone HI30, Biolegend, no. 304031 or 304036), anti-human CD3 (CD3, clone UCHT1, Biolegend no. 300429 or 300415), anti-human CD4 (CD4, clone OKT4, Biolegend no. 317447 or 317418), anti-human CD8 (CD8, clone RPA-T8, Biolegend no. 301045 or 301012), anti-human CCR7 (CCR7, clone 2-L1-A Biolegend no. 566762), anti-human CD62L (CD62L, clone DREG-56, BD Biosciences no. BDB565535), anti-human CD25 (CD25, clone BC96, Biolegend 302646) for 30 minutes and kept on ice. Samples were washed and then fixed and permeabilized with the FoxP3 transcription factor staining buffer set (eBiosciences no. 50-112-8857) following the manufacturer’s instructions. Intracellular staining was done with the following anti-human antibodies: FoxP3 (BD Biosciences, clone 236A/E7, no. BDB561493), T-bet (BD Biosciences, clone O4-46, no. BDB564141), GATA3 (BD Biosciences, clone L50-823, no. BDB563349) and RORγt (BD Biosciences, clone Q31-378, no. BDB562607) for 30 minutes and then samples were washed and resuspended in PBS. Samples were run on a LSR Fortessa flow cytometer (BD Biosciences) and analyzed with FlowJo software (BD Biosciences). Counting beads were added to blood samples to determine absolute counts of cell populations per microliter of blood. Graphs depict the frequency or counts of indicated cell types in each tissue. Gates were set using fluorescence minus one (FMO) controls for each tissue and were run with every experiment.

### Histopathology

Slide sections of day 31 livers were stained with hemoxylin and eosin for histological evaluation. A board-certified pathologist who was blinded to treatment groups scored liver sections for GvHD-related lesions on a scale of 0-11 including bile duct injury, inflammation and periportal necrosis, as described previously (20, 21). Graphs depict the total liver histopathological score for each mouse in each treatment group.

### Immunohistochemistry

Slides were incubated at 37°C for 20 minutes and then washed twice for 5 minutes in each of the following: Histo-clear (Fisher, no. 5089990147), 100%, 95%, 70%, 50% ethanol, and deionized water to remove paraffin wax and rehydrate tissues. Sections were then permeabilized in 0.2% Triton X-100 PBS for 15 minutes, washed with PBS and then antigen retrieval was done with warm 10 mM citrate buffer (Sigma, no. C9999) for 30 minutes. Slides were washed 3 times with PBS for 5 minutes and then blocked for 1 hour with 10% goat serum (Sigma, no. G9023). Sections were then incubated with anti-human CD45 rabbit IgG (1:200 in 1% BSA PBS, Cell Signaling Technologies, no. 13917S) overnight at 4°C in a humidified chamber. The next day, slides were washed with PBS thrice for 10 minutes and then incubated in AlexaFluor488-conjugated goat anti-rabbit IgG secondary antibody (1:500 in 1% BSA PBS, Thermo, no. A-11008) for 1 hour. Slides were again washed with three 10-minute PBS washes, submerged in water for 5 minutes and then counterstained with DAPI (1μg/mL) for 3 minutes. Slides were washed once for 10 minutes and then coverslips were mounted with Fluoromount G mounting medium (Fisher Sci, no. 5018788) and stored at 4°C. Slides of the same tissue and timepoint were imaged on the same day on a Leica DM5500 fluorescence microscope (Leica Microsystems, Wetzlar, Germany) with a 4-megapixel Pursuit camera (Diagnostic Instruments, Inc.) at 400x magnification using identical camera settings for all images. Human CD45 positive cells were quantified by manually counting the number of positive cells from at least 5 fields of view per mouse. Due to variability of human CD45 positive cell frequencies across the tissue in mice with increased infiltration, total positive cells were quantified from 10 fields of view to represent total tissue numbers more accurately. Total cell numbers were quantified using the automated Analyze Particles option using ImageJ software (NIH). Graphs depict the average number of human CD45 positive cells out of the total cell number for each individual mouse in each group.

### FITC-dextran assay

Mice were fasted for 4 hours to allow for intestinal clearance. Pre-gavage blood was collected into heparinized capillary tubes to account for background fluorescence variations between animals. Mice were then gavaged with 150uL of 80mg/mL FITC-dextran (Sigma, no. FD4) solubilized in sterile 1X PBS and after 4 hours, were bled again to determine the FITC content in blood. Plasma was collected and diluted 1:10 in PBS and loaded on a black 96-well microplate and fluorescence was read at 485/530n (ex/em) on a SpectraMax M3 microplate reader (Molecular Devices, San Jose, CA). Intestinal permeability is expressed as relative fluorescence units calculated as the difference between pre- and post-gavage blood and normalized to healthy NSG mice on each day of analysis.

### RNA extraction and bulk-mRNA sequencing

Spleens were collected and snap frozen with liquid nitrogen until further processing. Frozen spleens were homogenized in RLT buffer and RNA was isolated using the RNeasy mini kit (Qiagen, no. 74104) per the manufacturer’s instructions with the RNase-free DNase-kit (Qiagen no. 79254) and stored at -80°C. RNA samples were sent to Novogene for bulk mRNA sequencing. RNA was poly-A enriched, unstranded library prep was done using NEBNext Ultra II RNA library prep kit (New England Biolabs, Ipswich, MA) and then samples were sequenced on an Illumina NovaSeq PE150 (Illumina, San Diego, CA). Bioinformatics analysis was done by the University of Arizona Genomics Core. Reads were aligned to human (GRCh37) and mouse (GRCm26) genomes and disambiguated to distinguish human versus mouse reads using AstraZeneca’s disambiguate tool. Then, differential expression (DE) analysis was performed using edgeR. Low expressed genes were removed from the analysis using edgeR’s filterByExpr function. This resulted in 12,160 genes remaining for DE analysis. These remaining genes were also used to run gene set enrichment analysis (GSEA) against the KEGG database. The dotplot was made using ggplot. Only GSEA results with an FDR/q-value of *q<0.05* were incorporated into the dotplot. Heat maps were constructed using pheatmap on log transformed read count data using only genes considered core enriched by GSEA.

### Statistics

Statistical analysis was done using GraphPad Prism 9 software (La Jolla, CA). Survival and cumulative incidence data was analyzed using the Log-rank/Mantel-Cox test. Weekly average weight change and GvHD scores were evaluated with a two-way analysis of variance (ANOVA) with Šídák’s *post-hoc* comparisons. Differences between groups at a single timepoint were analyzed using a one-way ANOVA with Tukey’s *post-hoc* test for multiple comparisons or Dunnett’s *post-hoc* test when comparing to a control group, indicated in figure legends. A P-value of < 0.05 was considered statistically significant, with more significant values denoted by the number of symbols * <0.05, ** <0.01, ***<0.001, ****<0.0001. In some cases, asterisks (*) represent differences across all groups, whereas in others, varying use of symbols denote differences between specific treatment groups, which are listed in figure legends.

## Results

### Administration of CY/CY, BEN/BEN or CY/BEN improves survival from xGvHD compared to untreated mice while single day CY does not

**Figure 1:**
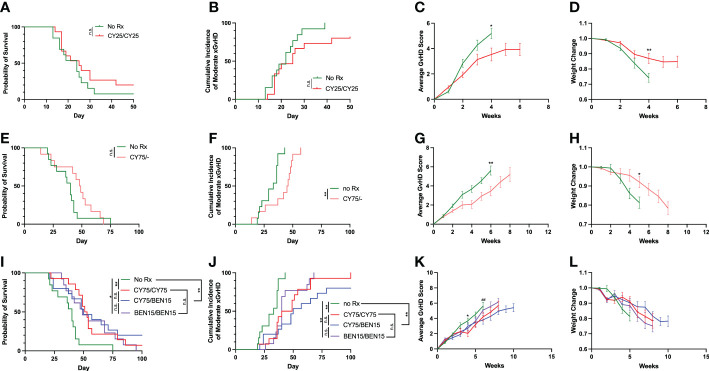
CY/CY, BEN/BEN or CY/BEN treatment improves survival and xGvHD severity, whereas lower dose or single day CY treatment is insufficient at improving survival when compared to untreated mice. Animals received 150 cGy irradiation on day -1, were infused with fresh-ficoll separated human PBMCs on day 0 and were treated with **(A-D)** 25mg/kg of CY on days +3 and +4 (CY25/CY25 n=15), or no pharmacological agent (no Rx n=13), **(E-H)** 75mg/kg CY on day +3 only (CY75/-, n=12), or no Rx controls (n=13), or **(I-L)** 75mg/kg of CY on days +3 and +4 (CY75/CY75, n=14), 15 mg/kg BEN on day +3 and +4 (BEN15/BEN15, n=13), 75mg/kg CY on day +3 and 15mg/kg BEN on day +4 (CY75/BEN15, n=15), or no Rx controls (n=13). **(A, E, I)** Kaplan-Meier survival curves of animals receiving various treatment regimens. **(B, C, F, G, J, K)** A clinical GvHD scoring system evaluating differences in activity, fur, posture, skin, and weight changes on a scale of 0-2 was used and assessed twice weekly throughout the study, with a possible total score range of 0-10. **(B, F, J)** Animals receiving a score of 5+ on two consecutive days or death of an animal is marked as an incidence of moderate xGvHD, depicted on graphs. **(C, G, K)** Average combined xGvHD scores. **(D, H, L)** Average weight change over time. **(A-L)** Data are combined from 3 independent experiments using different blood donors for PBMC infusion, with 12-15 total mice per group. **(A, B, E, F, I, J)** The Log-rank Mantel-Cox test for survival/incidence curves was used for statistical analysis, with p values indicated by the number of asterisks between groups: *<0.05, **<0.01, n.s.= not significant. **(C, D, G, H, K, L)** Statistics were run using a two-way ANOVA followed by Šídák’s multiple comparisons. P values are indicated by number of symbols between groups noted on graphs * 0.05, **<0.01. **(K, L)** Asterisks (*) indicate differences between no Rx and CY75/CY75. Pound symbols (#) indicate differences between no Rx and CY75/BEN15.

We initially performed experiments evaluating whether a 25mg/kg dose of CY on two days was sufficient for reducing xGvHD and improving survival, as this dose has previously been suggested as the optimal dose of CY for limiting GvHD in a haplo-BMT mouse model (9). The development of xGvHD was evaluated using a clinical scoring system on a scale of 0-2 for the following criteria: activity, fur, posture, skin, and weight loss (22). When an animal reached a total score of 5+ at two consecutive scoring days, they were considered as reaching moderate xGvHD. Mice treated with 25mg/kg of CY on days +3 and +4 had modest improvements in average xGvHD scores (**Figure 1C** *P<0.05*) and weight change (**Figure 1D**, *P<0.05)* but had similar rates of moderate xGvHD (**Figure 1B**, *P=0.10*) and survival (**Figure 1A**, *P=0.25*) compared to untreated controls, indicating that this dose does not adequately control xGvHD lethality. We therefore titrated up the dose of CY to 75mg/kg given on day +3 alone and found similar results of modest improvements in xGvHD and weight but no improvement in survival (**Figures 1E–H**). We next investigated whether two days of CY (75mg/kg), or BEN (15mg/kg) or the combination of the two agents given on days +3 and +4 would more effectively suppress xGvHD. We found that CY/CY, BEN/BEN and CY/BEN suppressed moderate xGvHD (**Figure 1J**, CY/CY vs. no Rx, *P=0.002*; BEN/BEN vs. no Rx, *P=0.003*; CY/BEN vs. no Rx, *P=0.002*) leading to significantly prolonged survival when compared to untreated mice (**Figure 1I**, vs. CY/CY, *P=0.002*; vs. BEN/BEN, *P=0.015*; vs. CY/BEN *P=0.009*) but with no difference between treated groups. Averages of xGvHD scores over time showed modest improvements with CY/CY and CY/BEN when compared to no Rx (**Figures 1K, L**, *P<0.05)*. We also evaluated whether a single higher dose of CY (150mg/kg) or BEN (30mg/kg) given on day +3 alone could mitigate xGvHD lethality in a similar fashion to lower doses over two days. Single-administration of higher doses of both chemotherapeutics was able to modestly improve survival, xGvHD and weight compared to no Rx (**Supplemental Figure 1**), but due to the current clinical practice of two day administration, the lower doses on days +3 and +4 were used for subsequent evaluation. Combined, these data illustrate that CY/CY, BEN/BEN or CY/BEN given post-PBMC infusion induce comparable protection against xGvHD morbidity and mortality.

### Administration of CY/BEN + CSA improves xGvHD and survival compared to CY/CY + CSA

**Figure 2:**
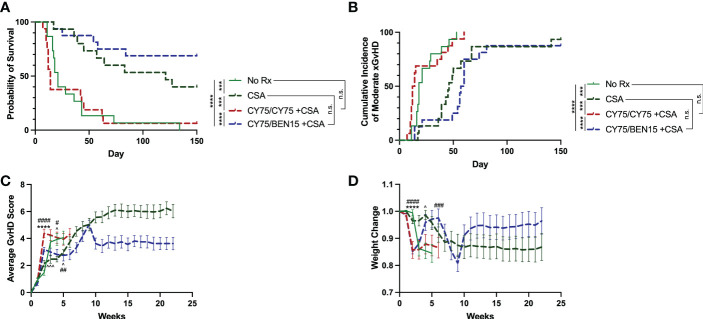
Survival from xGvHD is improved with combination CY/BEN +CSA. Animals received no pharmacological treatment (no Rx, n=15), or were treated with 10mg/kg daily cyclosporine A (CSA) for two weeks starting at day +5 and then 3x/week for the remainder of the study either alone (CSA, n=15), or in combination with post-PBMC infusion chemotherapy agents, 75mg/kg CY on days +3 and +4 (CY75/CY75 +CSA, n=16), or 75mg/kg CY on day +3 and 15mg/kg BEN on day +4 (CY75/BEN15 +CSA, n=16). **(A)** Kaplan-Meier survival curve illustrating differences in survival between groups. **(B)** Cumulative incidence of moderate xGvHD are depicted. **(A, B)** The Log-rank Mantel-Cox test for survival/incidence curves was used to assess statistical differences, with p values indicated by the number of asterisks between groups: ***<0.001, ****<0.0001, n.s.= not significant. **(C)** Average GvHD scores over time. **(D)** Changes in weight over time. **(C, D)** Statistics were run using a two-way ANOVA followed by Šídák’s multiple comparisons. P values are indicated by the number of symbols between groups noted on graphs *<0.05, **<0.01, ***<0.001, ****<0.0001. Arrows (^) indicate differences between no Rx and CSA. Asterisks (*) indicate differences between no Rx and CY75/CY75 +CSA. Pound symbols (#) indicate differences between no Rx and CY75/BEN15 +CSA. **(A-D)** Data are combined from 3 independent experiments using different blood donors for PBMC infusion with 15-16 mice total per group.

Since in most clinical regimens PT-CY is followed by a CNI, tacrolimus or cyclosporine A (CSA), we next aimed to evaluate whether the addition of CSA would further augment protection against xGvHD. PT-CY has become a standard GvHD prophylaxis regimen used clinically in haplo-HCT and, since we are evaluating the safety of utilizing PT-CY/BEN compared to PT-CY in our ongoing phase I trial (19), we therefore, compared the efficacy of these two treatment regimens in combination with CSA (CY/CY +CSA, CY/BEN +CSA) in our xGvHD model using doses of 75mg/kg of CY and 15mg/kg of BEN. In this model CSA alone had a significant effect in controlling xGvHD compared to untreated controls (**Figure 2**, *P=0.0001* survival*, P=0.0006* moderate xGvHD). Surprisingly, CY/CY +CSA failed to protect against xGvHD compared to untreated controls (**Figure 2**, *P=0.72* survival*, P=0.59* moderate xGvHD) and fared worse than mice receiving CSA alone (**Figure 2**, *P=0.0005* survival*, P=0.0008* moderate xGvHD). CY/BEN +CSA treated animals demonstrated significantly improved xGvHD morbidity and mortality compared to no Rx (**Figure 2**, *P<0.0001* survival*, P<0.0001* moderate xGvHD), CY/CY +CSA (**Figure 2**, *P<0.0001* survival*, P<0.0001* moderate xGvHD) and trended toward improved survival over CSA alone (**Figure 2**, *P=0.15*) but had no difference in moderate GvHD (*P=0.29*). CY/BEN +CSA treated mice did exhibit the lowest average GvHD scores and maintained the highest proportion of starting weight over time (**Figures 2C, D**). These data illustrate that post-PBMC infusion CY/BEN +CSA is safe and, in this model system, more effective at dampening xGvHD development when compared to CY/CY +CSA.

### CY/CY +CSA treated animals have reduced plasma albumin and increased intestinal permeability

**Figure 3:**
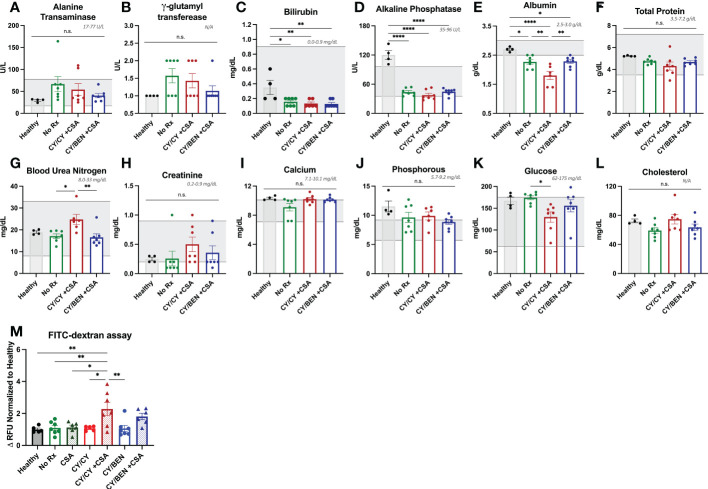
Combination CY/CY +CSA treated animals have significantly reduced plasma albumin and increased intestinal permeability at day 10 post-PBMC infusion. **(A-L)** Plasma was collected at day 10 from healthy untreated NSG mice (healthy), NSG mice that were conditioned with 150 cGy of radiation, infused with PBMCs and received no pharmacological agents (no Rx), or those treated with CY/CY +CSA or CY/BEN +CSA. A comprehensive metabolic panel was performed to evaluate potential toxicities from treatment or metabolic alterations indicative of GvHD. Gray numbers and shaded gray areas illustrate the normal range for mice of each given analyte on each graph. **(M)** Plasma was isolated from fasted mice before and 4-hours-post oral gavage of FITC-dextran to evaluate intestinal integrity. Values were calculated as a change in relative fluorescence units (RFU) and were normalized to the average values of healthy NSG mice on each day of analysis. **(A-M)** A one-way ANOVA with Tukey’s post-hoc comparisons was used to determine significant differences between treatments. P values are indicated by the number of asterisks between groups noted on graphs, *<0.05, **<0.01, ****<0.0001, n.s.= not significant.

Given the unexpected finding of lack of prevention of xGvHD with CY/CY +CSA, we performed a metabolic panel to discern whether this was due to organ toxicity from the therapy versus hyper acute xGvHD. Plasma was collected from mice of indicated groups at day 10 following PBMC infusion as well as from healthy NSG mice receiving no radiation or PBMC infusion. The concentration of albumin in the plasma was significantly reduced in all PBMC-infused groups compared to healthy NSG mice. Notably, the CY/CY +CSA treated group had significantly lower plasma albumin levels than CY/BEN +CSA (**Figure 3E**, *P=0.0059*) or untreated mice (*P=0.0075*), which has been identified as a potential biomarker for severe (grade 3-4) acute GvHD development in patients undergoing allo-HCT (23, 24). Transaminases and bilirubin that may indicate liver xGvHD if elevated, were unremarkable (**Figures 3A, C**). Blood urea nitrogen was modestly elevated in CY/CY +CSA plasma when compared to CY/BEN +CSA (**Figure 3G**, *P=0.0078*) or untreated (*P=0.0136*) mice, which may be indicative of dehydration from diarrhea. All other metabolic items evaluated were not significantly different between PBMC infused groups (**Figure 3B, D, F, H–L**). Lastly, to determine intestinal integrity as a measure of gut GvHD, an oral FITC-dextran challenge was performed to evaluate intestinal leakage into the blood. Mice receiving CY/CY +CSA combination treatment had significantly increased FITC-dextran content in plasma compared to all other groups (**Figure 3M**, *P<0.05*), with the exception of CY/BEN +CSA, which itself was not different from any other group. To further evaluate the potential toxicity of the drug combinations, we evaluated survival and weight change of animals that did not receive PBMC infusion but were given CY/CY +CSA or CY/BEN +CSA with and without irradiation on day -1. There was no difference in survival of mice across groups (**Supplemental Figure 2A**), but a modest reduction of weight was seen in both irradiated groups at days 7-11 (**Supplemental Figure 2B**, *P<0.05*), however, this would be expected to occur following total body irradiation, as there may be mild damage to intestinal tissue that could lead to weight loss. The changes in weight in the irradiated groups did rebound to similar levels in both non-irradiated groups by day 25 (**Supplemental Figure 2B**). Combined, these data illustrate the combination of therapeutic agents used in these studies did not induce toxicity in mice that would account for the mortality depicted in **Figure 2A** following CY/CY +CSA. Additionally, GvHD development in CY/CY +CSA treated mice is severe at day 10, supported by reduced plasma albumin and increased intestinal permeability.

### Animals treated with chemotherapy with or without CSA have reduced human CD45, CD4 and CD8 T cell numbers early after PBMC infusion with sustained reductions in CD4 T cells at day 31

**Figure 4:**
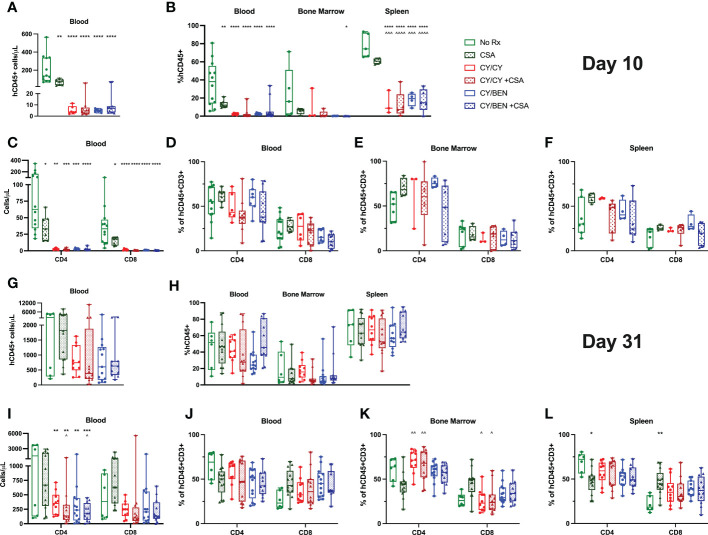
Post-PBMC infusion chemotherapy with or without CSA significantly reduces total human CD45+ cells and T cells at day 10 with a sustained reduction in circulating absolute CD4 T cell counts at day 31. PBMC-infused mice were given indicated treatments and blood, bone marrow, and spleens were collected for analysis on **(A-F)** day 10 or **(G-L)** day 31. **(A, C, G, I)** Absolute numbers were calculated per microliter of blood using counting beads for **(A, G)** total CD45+ or **(C, I)** CD4+ or CD8+ T cells. **(B, H)** Percentage of human CD45+ cells out of total cell numbers in blood, bone marrow and spleen. **(D-F, J-L)** percentages of CD4 and CD8 T cells in **(D, J)** blood, **(E, K)** bone marrow and **(F, L)** spleen. **(A-L)** Significant differences were determined via a one-way ANOVA followed by Tukey’s post-hoc comparisons. P values are indicated by the number of symbols between groups noted on graphs, *<0.05, **<0.01, ***<0.001, ****<0.0001. Asterisks (*) indicate significant differences from no Rx, arrows (^) indicate differences from CSA group.

Blood, bone marrow and spleens were collected from mice at days 10 and 31 following PBMC infusion for flow cytometric analysis and human CD45 positive absolute cell numbers were calculated with the use of counting beads. At day 10, untreated mice had significantly higher human CD45 counts in blood compared to CSA (**Figure 4A**, *P<0.01*), CY/CY, or CY/BEN groups with or without CSA (*P<0.0001*), which correlated with the percentages of human CD45 positive cells seen in the blood and spleen at day 10 (**Figure 4B**). Overall, human CD45 percentages of bone marrow stayed relatively low over time (**Figures 4B, H**). By day 31, human CD45 counts (**Figure 4G**), and frequencies (**Figure 4H**) were similar across all treatment groups. Therefore, while there is a delay in human CD45 cell counts and frequencies at early timepoints in CY/CY or CY/BEN treated groups, donor hematopoietic cell reconstitution equilibrates across treatment groups by day 31. CD4 and CD8 T cell subsets were also evaluated in lymphoid organs. Absolute CD4 and CD8 T numbers were significantly reduced in all pharmacological treated groups compared to untreated controls at day 10 (**Figure 4C**, *P<0.05*), whereas their frequencies were not found to be different between groups in blood, bone marrow or spleens (**Figures 4D–F**). At day 31, counts of CD4 T cells were significantly reduced in CY/CY and CY/BEN groups with or without CSA when compared to untreated mice (**Figure 4I** *P<0.01*). Additionally, CY/CY +CSA and CY/BEN +CSA groups showed significantly reduced CD4 T cells compared to CSA only mice (**Figure 4I**, *P<0.05*). Frequencies of CD4 and CD8 T cells were similar across blood, bone marrow and spleen at day 31 (**Figures 4J–L**), with the exception of CSA only treated mice that showed a slight reduction of CD4 and increase of CD8 T cell frequencies in bone marrow compared to CY/CY groups (**Figure 4K**, *P<0.05*) and in spleen when compared to no Rx (**Figure 4L**, *P<0.05*). Taken together, we show delayed immune recovery in all CY and/or BEN treated groups that reach similar levels to untreated by day 31, with the exception of absolute counts of CD4 T cells that remain significantly lower in blood.

### Post-PBMC-infusion chemotherapy treatment reduces human immune cell infiltration into target organs and GvHD-related lesions in the liver

**Figure 5:**
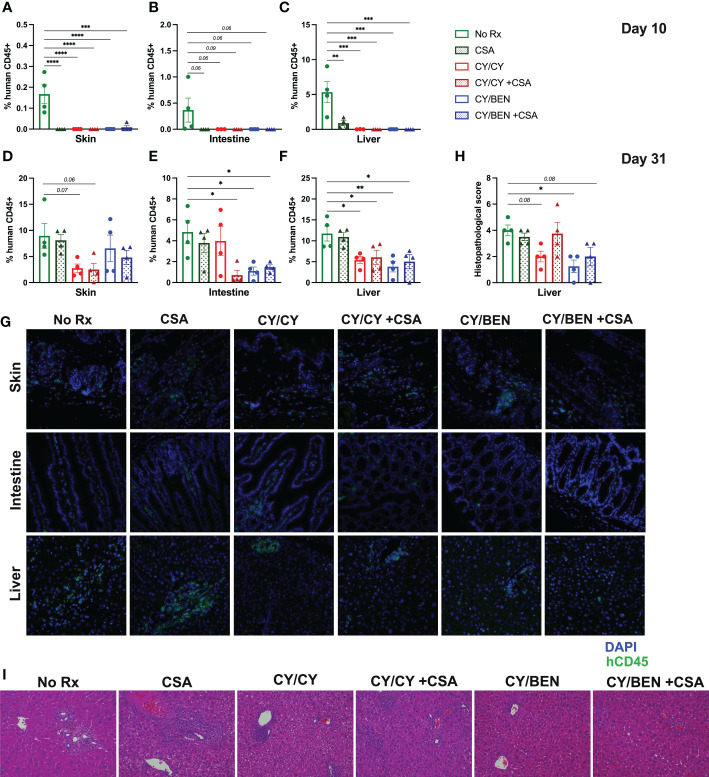
Histopathological alterations and human immune cell infiltration of GvHD target organs is reduced at days 10 and 31 with post-PBMC chemotherapy use with or without the addition of CSA. **(A-G)** Immunohistochemistry of human CD45+ cells in skin, intestine and liver sections from mice in each treatment group was performed at **(A-C)** day 10 and **(D-G)** day 31 post-PBMC infusion to evaluate the extent of immune infiltration of GvHD target organs. **(A-F)** Average percent of human CD45+ cells out of the total cell number in **(A, D)** skin, **(B, E)** intestine and **(C, F)** liver. **(G)** Representative images of tissues from animals in each treatment group at day 31. **(H, I)** Liver tissue sections were stained with hematoxylin and eosin and scored blindly by a board-certified pathologist for GvHD-related lesions. **(H)** Average scores of liver lesions per treatment group. **(I)** Representative images of liver sections from animals in each treatment group. **(A-F, H)** Significant differences from no Rx was evaluated using a one-way ANOVA and Dunnett’s post-hoc comparisons with P values indicated by the number of asterisks between groups noted on graphs, *<0.05, **<0.01, ***<0.001, ****<0.0001.

At days 10 and 31 following human PBMC infusion, skin, intestine, and liver tissues were collected for histological analysis. Infiltration of human immune cells into target organs was evaluated *via* immunohistochemistry for human CD45 positive cells in each tissue and was calculated as a percentage of the total cell number. At day 10 following PBMC infusion, all mice receiving pharmacological treatment show a reduction in liver and skin infiltration of human CD45 positive cells when compared to untreated mice (**Figures 5A, C**, *P<0.01*) that trended towards significant in intestine (**Figure 5B**), albeit percentages are quite low in all groups at this timepoint, particularly for intestine and skin. Unexpectedly, rates of human CD45 cells in the skin were not significantly reduced at day 31 but trended towards lower in CY/CY treated animals with or without CSA (**Figure 5D**). Intestinal human CD45 cell infiltration was reduced in CY/CY +CSA, CY/BEN, and CY/BEN +CSA groups when compared to untreated mice (**Figure 5E**, *P<0.05*) but not in those treated with CY/CY. At day 31, there was significant infiltration of human CD45 positive cells in the liver of untreated mice that was not different from animals treated with only CSA but was significantly reduced with post-PBMC chemotherapy (**Figure 5F**, *P<0.05*). To further assess differences in histopathology between groups, liver sections from mice in each treatment group were blindly scored by a board-certified pathologist for GvHD-related lesions as previously described (20, 21). Briefly, livers were scored for bile duct injury, inflammation and periportal necrosis on a scale of 0-11 and the combined tissue score is illustrated on graphs. Mice receiving CY/BEN have significantly lower liver histopathology scores when compared to untreated control mice (**Figures 5H, I**, *P<0.05*), with CY/BEN +CSA and CY/CY groups trending towards lower liver GvHD scores. Together, these data illustrate that the use of post-PBMC chemotherapy significantly reduces immune infiltration of target organs, thus limiting the development of more aggressive GvHD.

### Post-PBMC CY/BEN with or without CSA reduces circulating numbers and frequencies of naïve and effector memory T cells

**Figure 6:**
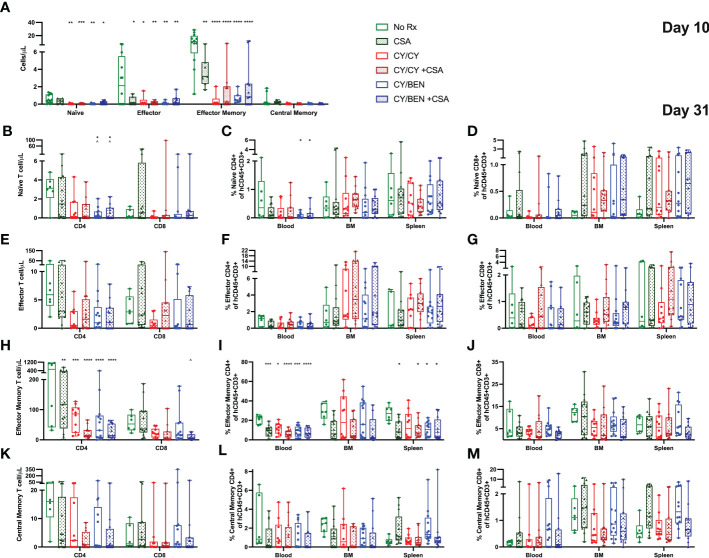
Post-PBMC infusion CY/BEN with or without CSA significantly reduces the absolute counts and frequencies of naïve and effector memory T cells at days 10 and 31. Blood, bone marrow and/or spleens were collected from animals at days 10 and 31 post-PBMC infusion and prepared for flow cytometric analysis as described in Materials and Methods. **(A)** Absolute counts of circulating effector T cell subsets at day 10, gated as described below. **(B-M)** Day 31 frequencies and counts of effector T cell subsets. All subsets were gated from CD45+CD3+ cells **(B-D)** Naïve T cells were gated as CCR7+CD62L+CD45RA+. **(E-G)** Effector T cells were gated as CCR7-CD62L-CD45RA+. **(H-J)** Effector Memory cells were gated as CCR7-CD62L-CD45RO+. **(K-M)** Central Memory T cells were gated as CCR7+CD62L+CD45RO+. **(A-M)** Significant differences across treatment groups were determined via a one-way ANOVA followed by Tukey’s post-hoc comparisons. P values are indicated by the number of symbols on graphs, *<0.05, **<0.01, ***<0.001, ****<0.0001. Asterisks (*) mark significant differences from vehicle group, arrows (^) mark differences from CSA group.

We next aimed to evaluate potential differences in effector and memory T cell subsets across lymphoid tissues at days 10 and 31. At day 10, absolute counts of total effector and effector memory T cells were significantly reduced in blood of groups treated with any pharmacological agent post-PBMC infusion (**Figure 6A**, *P<0.05*), with CY/CY and CY/BEN groups also showing a reduction in circulating counts of naïve T cells (*P<0.05*). Of note, the low cell numbers in blood, bone marrow and spleen observed at day 10 (**Figures 4A–C**, **Figure 6A**) limit the reliable detection of rarer cell populations; therefore, additional analysis at this timepoint was limited to ensure reliability of the presented data. At day 31, there was a reduction in the counts (**Figure 6B**, *P<0.05*) and frequency (**Figure 6C**, *P<0.05*) of naïve CD4 T cells in blood of CY/BEN treated mice with or without CSA. Additionally, there is a significant reduction in CD4 T effector memory cell frequencies in blood and spleens of animals receiving any post-PBMC treatment, with the exception of CY/CY treated spleens (**Figure 6I**, *P<0.05*), which correlates with reduced circulating counts of effector memory CD4 T cells in all post-PBMC chemotherapy treated mice (**Figure 6H**, *P<0.01*). There were no changes in CD4 central memory or effector T cells or in any CD8 effector or memory T cell subset at day 31 (**Figures 6D–G, J–M**). The gating strategy used for these analyses is illustrated in **Supplemental Figure 3** and was determined with the use of FMO controls that were run with each experiment. Further evaluation of undefined populations of T cells that may be transitioning between effector/memory phenotypes, as denoted by varying presence of CCR7 and CD62L, showed no statistical difference across any group (**Supplemental Figure 4**). The combination treatment with BEN may have an additive effect on GvHD control by reducing naïve CD4 T cell numbers and frequencies in addition to the reduction of CD4 effector memory T cells seen with CY or other post-PBMC pharmacological agents presented here and previously shown in other murine GvHD models (25, 26).

### Post-PBMC treatments differentially regulate T helper-17 inflammatory and T regulatory cell frequencies in lymphoid tissues

**Figure 7:**
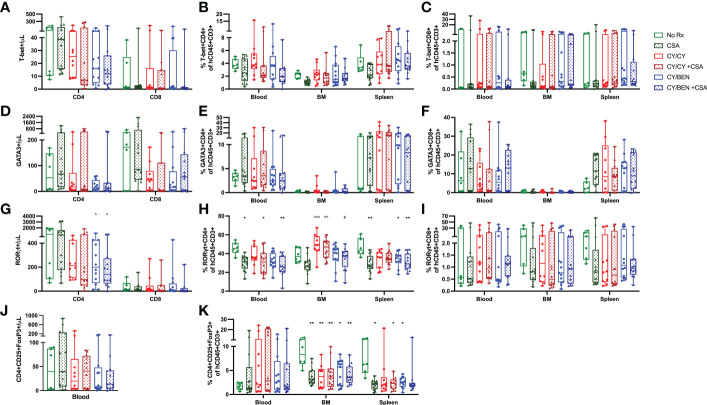
T helper 17 inflammatory T cell polarization and T-regulatory cell percentages are modestly different at day 31 with post-PBMC chemotherapy treatment. Blood, bone marrow and spleen were collected from animals at day 31 post-PBMC infusion and prepared for flow cytometric analysis as described in Materials and Methods. All subsets were gated from CD45+CD3+ cells and identified as specific T helper subsets via the presence of their respective transcription factors: **(A-C)** T helper 1 cells: T-bet+. **(D-F)** T helper 2 cells: GATA3+. **(G-I)** T helper 17 cells: RORγt+. **(J, K)** Regulatory T cells: CD4+CD25+FoxP3+. **(A, D, G, J)** Absolute counts of each T helper subset present in blood were determined with the use of counting beads. **(A-K)** Significant differences across treatment groups were evaluated via a one-way ANOVA followed by Tukey’s post-hoc test. P values are indicated by the number of symbols between groups, *<0.05, **<0.01, ***<0.001. Asterisks (*) indicate significant differences from vehicle group, arrows (^) indicate differences from CSA group, and pound symbols (#) indicate differences from CY/CY group.

To measure differences in T helper -1, -2 and -17 cells (Th1, Th2, Th17, respectively), blood, bone marrow and spleens were collected from animals at day 31 post-PBMC infusion and prepared for flow cytometric analysis. T helper cell types are marked by the presence of specific transcription factors; Th1 cells are identified *via* T-bet expression, Th2 are identified *via* GATA3, and Th17 are identified *via* RORγt (27). The gating strategy used for identifying these populations is outlined in **Supplemental Figure 5**, with all gates being placed using FMO controls for each tissue and run with each experiment. Th1 and Th2 polarized T cell frequencies were not different across treatments in any tissue evaluated, nor were circulating counts (**Figures 7A–F**). Th17 cell frequencies were significantly lower in blood of CSA treated groups (CSA, CY/CY +CSA, and CY/BEN +CSA, **Figure 7H**, *P<0.05*); however, absolute counts of Th17 cells in blood were not different from untreated mice (**Figure 7I**). CY/BEN mice with or without CSA showed a modest reduction in absolute numbers of circulating Th17 cells when compared to CSA only treated mice (**Figure 7G**, *P<0.05*), whereas CY/CY treated animals with or without CSA had significantly more Th17 T cells in bone marrow when compared to CSA (**Figure 7H**, *P<0.01*). Interestingly, splenic Th17 T cell frequencies were reduced in CSA, CY/BEN, and CY/BEN +CSA (**Figure 7H**, *P<0.05*) groups. Combined, the alterations in Th17 cells are inconsistent but suggest a possible influence of CSA and/or BEN in influencing proportions of Th17 polarized cells that may slow GvHD progression. T regulatory cell (Tregs) frequencies and absolute counts (**Figures 7J, K**) were not found to be different between groups in blood but were significantly reduced in the bone marrow of all mice treated with post-PBMC pharmacological agents (**Figure 7J**, *P<0.05*). Spleens of all treated mice also had reduced or trended toward lower frequencies of Tregs (**Figure 7K**, *P<0.05*). Therefore, treatment with CY, BEN or CSA impedes T regulatory cell expansion in bone marrow and spleen but did not appear to impact frequencies in circulation.

### Transcriptomes of spleens from mice at day 31 in CY/CY and CY/BEN groups show modest changes in migration, proliferation/differentiation, and inflammation pathways

**Figure 8:**
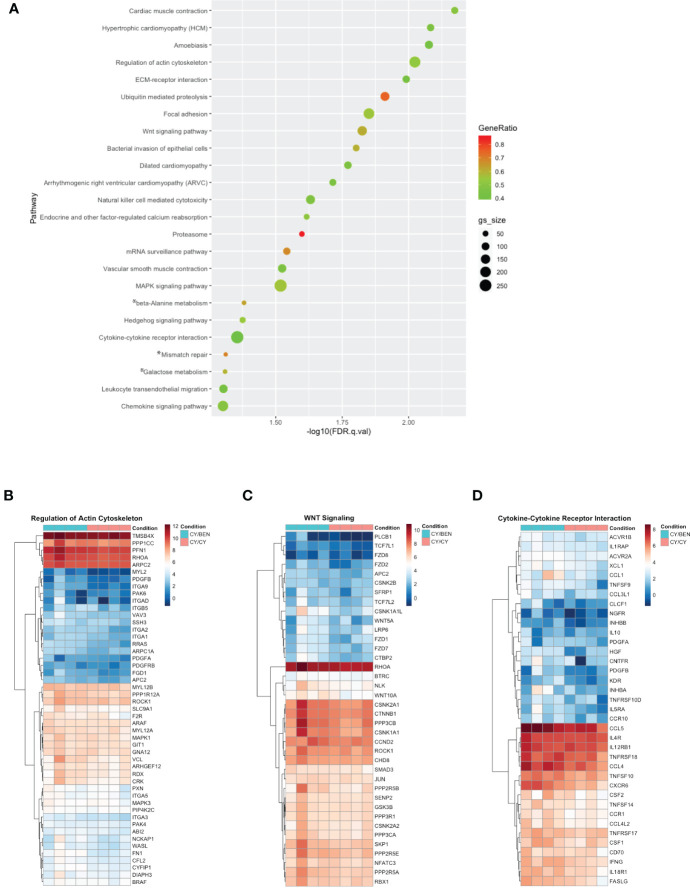
Gene set enrichment analysis annotated to KEGG terms reveals changes in multiple pathways related to cell migration, proliferation/differentiation, and inflammation between CY/CY and CY/BEN groups. **(A)** Dot plot of significantly different KEGG terms identified via gene set enrichment analysis from transcriptomes of spleens from CY/CY and CY/BEN treated mice at day 31 following PBMC infusion. GSEA results with a q-value of q<0.05 are shown. Asterisks indicate KEGG terms with normalized enrichment scores trending positive in CY/CY treated spleens. **(B-D)** Heat maps illustrating differences in GSEA genes associated with specific KEGG pathways were generated from log-transformed gene counts using only genes denoted as core enriched for **(B)** regulation of actin cytoskeleton, **(C)** Wnt signaling, and **(D)** cytokine-cytokine receptor interactions.

To avoid RNA degradation and reduce digestion-mediated alterations in RNA, whole spleens containing a mixture of murine and human cells were harvested from animals at day 31 post-PBMC infusion. Poly-A enriched RNA was used for library preparation, sequenced, disambiguated from mouse genes and mapped to the human reference genome. Differential gene expression and gene set enrichment analysis (GSEA) annotated to KEGG terms was compared between CY/CY and CY/BEN groups to identify chemotherapy-mediated changes in splenic transcriptomes. Comparisons between CSA treated groups were not used, as the addition of CSA masked changes observed between CY/CY and CY/BEN groups (not shown). Differences in gene sets enriched to a number of KEGG terms associated with cell migration were noted *via* pathway differences in regulation of actin cytoskeleton, ECM-receptor interactions, focal adhesion, chemokine signaling, and leukocyte transendothelial migration, which were decreased in CY/CY spleens (**Figure 8A, B**). Modulation of proliferation and differentiation pathways was also illustrated with changes in regulation of the actin cytoskeleton, Wnt signaling and Hedgehog signaling, which were reduced in CY/CY groups (**Figures 8A–C**). Lastly, a reduction in inflammatory pathways with CY/CY treatment was observed *via* changes in cytokine-cytokine receptor interaction, mitogen-activated protein kinase (MAPK) signaling, and chemokine signaling (**Figure 8A, D**). While numerous differences in KEGG terms are indicated, differences in gene sets associated with each term are modest and show variation across samples and within groups (**Figures 8A–D**), which is likely influenced by differences in blood donors, altered compositions of human lymphocytes in mouse spleens, and severity of GvHD at time of harvest. The top differentially expressed genes between groups shows similar variability across samples (**Supplemental Figure 6**). We also evaluated changes in gene sets between no Rx and CY/CY or CY/BEN treatments, which denoted alterations in oxidative phosphorylation, ribosome and Parkinson’s disease associated genes (**Supplemental Figure 7**), with striking similarities in altered genes seen in both treated groups. Additional transcriptome analysis with a wider variety of samples utilizing diverse blood donors may be more informative of major pathways that are altered with CY/CY versus CY/BEN treatment and may uncover novel mechanistic differences occurring between these treatment regimens.

## Discussion

The use of post-transplant CY, combined with calcineurin inhibitors and/or other immunomodulatory agents has become the standard of care for GvHD prophylaxis in haplo-HCT and has seen increasing use in other types of allo-HCT. However, as viral reactivation due to delayed immune reconstitution and relapse often lead to morbidity and mortality of patients (3, 6, 28–30), some investigators have begun evaluating a reduction in the dose of post-transplant CY to limit these complications (8, 10, 11). Our laboratory has taken a different approach in evaluating whether partially replacing PT-CY with post-transplant bendamustine (PT-BEN) would be advantageous, which is based on our extensive research that has delineated several immunomodulatory properties of BEN on myeloid derived suppressive cells (MDSCs) and dendritic cell (DC) subsets (14–16). Moreover, we have demonstrated that BEN yields tolerant T-cells with a striking absence of GvHD, while preserving T-cell dependent graft-versus-tumor (GvT) effects (12). Our preclinical murine studies have led to a Phase Ia/b clinical trial in haplo-BMT (17, 19). In the present study, we aimed to confirm the safety and efficacy of CY/BEN in a xGvHD model. We first evaluated the efficacy of a 25mg/kg dose of CY on days +3 and +4, as this was previously deemed the ideal dose for controlling GvHD development in a haplo-BMT model (9). We show that the 25mg/kg dose of CY was unable to control xGvHD lethality (**Figures 1A–D**). We also evaluated the potential benefit of a single dose of CY at 75mg/kg on day +3 only, which failed to improve survival compared to untreated controls (**Figures 1E–H**). This finding was unexpected, as previous groups have shown that a single dose of CY at 100mg/kg on day +3 is sufficient for reducing xGvHD lethality (31, 32), albeit the dose of human PBMCs was lower (2x10^6^) in the referenced study by Ehx et al. (2021), which may account for the differences seen (31). In the other study, NSG mice received an identical human PBMC dose (5x10^6^) and survival of CY treated mice was significantly improved compared to untreated (32) but both groups exhibited shorter overall survival than what was observed with mice presented here (**Figures 1E–H**). A higher pre-transplant irradiation at 2.5 Gy was used in the previous publication, which may have shortened time to death (32). We next confirmed in this xenogeneic model that BEN/BEN is comparable to CY/CY in protecting against xGvHD (**Figures 1I–L**) as we have reported in a murine haplo-BMT model (13). We also evaluated the addition of BEN on day +4 to CY on day +3 (CY/BEN) and found it to have a suppressive effect against xGvHD compared to no Rx and similar to CY/CY and BEN/BEN (**Figures 1I–L**). We also show that single higher dose of either CY or BEN given only on day +3 can mitigate xGvHD lethality (**Supplemental Figure 1**) but was less impressive than lower doses given over days +3 and +4 (**Figures 1I–L**) and is not the current clinical practice for GvHD prophylaxis, which is why the two-day administration model was used for continued evaluation. Combined, these data refute the suggestion that the advantage of our CY/BEN regimen in improving outcomes is not due to the addition of BEN per se but to a reduction in the total dose of CY.

We furthermore examined the effect of adding CSA treatment in this model. Mice treated with only CSA showed a significant reduction in xGvHD and improvement in survival (**Figure 2**). The addition of CY/BEN to CSA further suppressed xGvHD (**Figure 2**) whereas mice receiving CY/CY +CSA did worse than CSA alone and no better than untreated controls (**Figure 2**). It is not clear why the combination of CY/CY +CSA failed to improve morbidity and mortality from xGVHD (**Figure 2**). We found that this group had a decrease in serum albumin and an increase in intestinal permeability (**Figures 3E, M**) indicative of intestinal GvHD. Although immunohistology did not reveal major differences in human CD45 positive cell infiltration in GvHD target organs such as liver, intestine, and skin between CY/BEN and CY/CY groups, it did show improvements over those receiving CSA alone (**Figure 5**). Importantly, we show that without human PBMC infusion, mice given CY/CY +CSA or CY/BEN +CSA do not experience drug-related toxicity (**Supplemental Figure 2**), which further supports the observation that the differences in survival seen (**Figure 2**) are primarily due to GvHD.

We next aimed to evaluate whether CY/CY or CY/BEN treatment altered immune subsets following PBMC infusion. There were minimal differences in immune subsets or activation status of T cells in mice treated with CY/CY versus CY/BEN at early or late timepoints (**Figures 4**, **6**, **7**). As we would expect, there were reduced cell numbers of total human CD45 positive cells, CD4 and CD8 T cells at day 10 in CY/CY and CY/BEN treatment groups when compared to controls, although percentages were maintained within normal ranges (**Figures 4A–F**). At day 31, we saw a maintained reduction in total CD4 T cell counts in blood of CY/CY or CY/BEN treated mice versus untreated (**Figure 4I**) that correlated with reduced xGvHD (**Figure 2**). Reduced percentages and absolute counts of CD4 T cells following CY treatment has been noted in blood and spleen at days 7 and 21 post-BMT in a haploidentical mouse model, which was suggested to be a major contributing factor by which CY mediates GvHD progression (26). However, in our model, while absolute counts in blood were reduced, frequencies of CD4 T cells were not different in blood, bone marrow or spleen (**Figures 4J–L**). Other notable changes when compared to untreated mice were the significant reduction in counts of naïve, effector and effector memory T cells at day 10 (**Figure 6A**), with a sustained reduction in counts and frequencies of effector memory CD4 T cells at day 31 in blood and spleens of chemotherapy treated groups (**Figures 6H, I**). This partially aligns with recently published data showing that post-transplant CY reduced percentages of splenic effector/effector memory CD4 T cells at day 7 and 21 in a haplo-BMT model, although circulating percentages were not different from vehicle controls at day 21 in this setting (26). Mice treated with CY/BEN also had a reduction in circulating naïve T cells at day 31 (**Figures 6B, C**) which may be a unique mechanistic component by which BEN impacted GvHD development in this model. CY treated mice did not show changes in naïve T cell frequencies or counts at day 31 (**Figure 6**), which contrasts with a previous report showing an increase in naïve T cell percentages with PT-CY at days 7 and 21 (26), although this may be due to differences between use of a haplo-BMT mouse model versus our xenogeneic model. While naïve T cells are important for reducing rates of infections and inducing GvT effects (33), they have been shown to exacerbate GvHD development following allo-HCT. The presence of naïve T cells correlates with chronic GvHD development in patients undergoing allo-HCT (34) and depletion of naïve T cells from donor grafts has shown promise in reducing acute and chronic GvHD (35, 36). Therefore, the reduction of naïve T cells seen with CY/BEN treatment may allow for improved GvHD control.

Interestingly, all chemotherapy treated groups were found to have reduced percentages of Tregs in bone marrow and spleen, however, circulating numbers and frequencies were not different across treatments (**Figures 7J, K**). Previous studies have indicated that the presence of Tregs helps maintain donor immune cell tolerance to host organs to limit GvHD development (37), and depletion of Tregs from donor grafts abrogated the protective effects of PT-CY in a xenogeneic GvHD model (32). Therefore, with a reduction of Tregs, one would expect to see an increase in GvHD severity, but perhaps, the presence of Tregs in the blood, was sufficient for xGvHD control (**Figure 2**). Post-PBMC administration of CSA is known to limit Treg expansion (38), as CSA blocks NFAT-mediated IL-2 production, which is a critical factor for Treg differentiation (37). The use of other immunomodulatory agents, such as rapamycin that does not hinder but rather enhances Treg expansion (31, 38, 39) may be a preferred alternative over CSA for GvHD prophylaxis. Previous data illustrates the safety and efficacy of rapamycin given alone or in combination with CY for mitigating xGvHD, which correlated with elevated Treg numbers in treated mice (31), making evaluation of rapamycin in combination with BEN a critical future direction. Interestingly, Tregs have previously been shown to be resistant to CY-mediated death due to upregulation of aldehyde dehydrogenase (32), a well-established group of enzymes that metabolize CY, with increased levels shown to induce CY resistance in various cancers (40, 41). In this study we show a reduction of Tregs in all treatment groups, including in animals treated only with CY, albeit our Treg analysis was limited when compared to that used in the previous report (32). Additional research evaluating mechanistic differences of CY/CY versus CY/BEN with regards to human T cell expansion or deletion is needed.

Polarization of T cells into helper -1, -2, or -17 phenotypes influences their function and has been shown to impact GvHD development, especially Th1 and Th17 polarized cells (42). Th1 polarization is identified *via* increased expression of the transcription factor T-bet, whereas Th17 cells upregulate RORγt (27). It was previously believed that Th1 polarized cells were major contributors of GvHD development (43), with many studies showing Th1 cells correlating with increased GvHD (44–46). In our study, frequencies of inflammatory T cell subsets inconsistently varied across organs and treatments, with no difference in Th1 or Th2 subsets across treatments (**Figure 7**). Unexpectedly, the level of Th1 polarized cells was quite low in this model with our analysis when compared with previously published work by other groups with Th1 cells comprising 60-80% of total T cells (45, 47). However, the methodology used to identify Th1 cells in these studies was cytokine-based rather than transcription factor based, which may be why more Th1 cells were captured in these publications (45, 47). Our findings do suggest that BEN and CSA may influence Th17 cell polarization in spleen and blood (**Figure 7**). Previous studies have presented conflicting results regarding the role of IL-17 and Th17 inflammatory T cells in GvHD but in general, their presence appears to exacerbate GvHD (42). When *in vitro* Th17 polarized cells were added to donor grafts, mice exhibited amplified GvHD severity in a major MHC mismatched model, with mice infused with Th1 polarized cells unexpectedly having improved survival over Th17 recipients (48). Similar findings have also been illustrated in a haplo-BMT model, a minor MHC mismatch model (49) and a xenogeneic model (50), with a particular increase in skin GvHD noted in multiple models following Th17 infusion (49, 50). In a xenogeneic model with NSG mice, treatment with CTLA-4 immunoglobulin to block T cell co-stimulation was shown to impede GvHD development and spleens isolated from mice had reduced gene expression of Th17 differentiation (44), which agrees with our findings presented here. A better understanding of how BEN influences T cell polarization, especially Th17 polarization, may uncover new insights into mechanisms of GvHD prevention.

Comparisons of transcriptional alterations induced by CY versus BEN has not been previously evaluated, particularly in the context of post-transplant GvHD prophylaxis. Previous studies have investigated transcriptomic alterations induced by CY in various tumor models (51, 52), whereas BEN-mediated changes have primarily been evaluated in the context of tumor resistance to therapy (53). CY administration every 6 days in a syngeneic C57BL/6 mouse glioma model demonstrated that CY modulates a variety of immune-mediated pathways similar to what we observed in our xGvHD model including, cell adhesion/focal adhesion, chemokine signaling, cytokine-cytokine interactions, and JAK/STAT signaling, and was also shown to down regulate steroid biosynthesis, cell cycle, and DNA replication (52). Further, glioblastoma cells treated with a CY metabolite illustrate modulation of mitophagy, MAPK signaling, and Akt survival pathways (51), many of which we found to be different between CY/CY and CY/BEN treated mice. BEN-mediated transcriptomic alterations have primarily been evaluated in the context of tumor resistance to therapy, and ingenuity pathway analysis revealed alterations in cell proliferation, signaling, survival and motility, which differed between BEN responsive versus resistant tumors (53). CY and BEN are both alkylating agents known to induce DNA damage and, thus, limit cell proliferation, whereas BEN can also act as a purine analog, allowing for anti-metabolite properties that augment the anti-proliferative effect (54, 55). Therefore, one may postulate there would be major alterations in transcriptomes of immune cells isolated from CY/CY versus CY/BEN treated mice. In the current study, RNA sequencing analysis of day 31 splenic transcriptomes suggests similar alterations between no Rx and CY/CY or CY/BEN (**Supplemental Figure 7**). When evaluating differences between treatments, the combination of CY/BEN was shown to differentially modulate expression of genes associated with cell migration, proliferation/differentiation, and inflammation when compared to CY/CY due to differences in gene sets enriched to multiple KEGG terms (**Figure 8**). Cell migration is influenced by actin cytoskeletal regulation, extracellular matrix-receptor interactions, and changes in focal adhesion and is often mediated by migratory-stimulating factors such as chemokines (56), all of which were found to be downregulated in CY/CY spleens when compared to CY/BEN. Additionally, a reduction in leukocyte transendothelial migration was noted with CY/CY. Cell proliferation/differentiation pathways were also reduced in CY/CY spleens, illustrated by changes in actin cytoskeletal regulation, of which upregulation is required to allow for cell division, coinciding with upregulation of Wnt and Hedgehog signaling in CY/BEN spleens. Wnt (57) and Hedgehog (58) signaling pathways are essential mediators of proliferation and differentiation of various cell types, including hematopoietic stem/progenitor cells (HSC) (59, 60) and T cells (61, 62). Therefore, increased gene set enrichment of these KEGG terms in CY/BEN treated spleens may indicate improved HSC proliferation and subsequent T cell differentiation, which could influence the downstream graft-versus-tumor effect that is critical for the success of allo-HCT in controlling hematological malignancies. We have previously shown that mice treated with BEN either as a pre-transplant conditioning regimen (12) or when given post-transplant in a haplo-BMT model (13) have improved GvT when compared to those treated with CY. Further research evaluating the ability of CY/CY versus CY/BEN treated grafts to control leukemia in a xenogeneic model, and the potential influence of Wnt or Hedgehog pathways in this response is warranted.

In summary, using another model, in this case xGvHD in NSG mice, we confirm our previous findings that BEN has comparable effects to CY in suppressing xGvHD morbidity and lethality (12, 13, 15–19, 55, 63). Moreover, combining CY with BEN appears to have advantages in improving survival from xGvHD which is not due to simply reducing the CY dose. Findings presented here and in previous publications evaluating BEN in haplo-BMT (12, 13, 15–19, 55, 63) may be applicable to other types of allo-HCT, such as unrelated HLA-matched or mismatched HCT, G-CSF mobilized peripheral blood stem cell grafts, or others, and supports the notion of further evaluating BEN as a GvHD prophylaxis therapy. Additional research delineating mechanistic differences between CY and BEN is necessary to fully uncover the potential advantages of BEN in the post-transplant setting.

## Data availability statement

The datasets presented in this study can be found in online repositories. The names of the repository/repositories and accession number(s) can be found below: https://www.ncbi.nlm.nih.gov/, PRJNA876310.

## Ethics statement

The animal study was reviewed and approved by University of Arizona Institutional Animal Care and Use Committee.

## Author contributions

KG, DD, and EK conceptualized and designed experiments. KG, MC, AM, and DD conducted experiments. KG, RS, and EK interpreted data. KG curated and visualized data. KG drafted the manuscript. EK and RS critically evaluated the manuscript. All authors contributed to the article and approved the submitted version.
