# Supplementary material for: Partially replacing cyclophosphamide with bendamustine in combination with cyclosporine A improves survival and reduces xenogeneic graft-versus-host-disease
Source: Front Immunol. 2023 Jan 9;13:1045710. doi: 10.3389/fimmu.2022.1045710 (PMC9868157; doi:10.3389/fimmu.2022.1045710)
Supplement: Supplementary file 1 [file DataSheet_1.pdf]

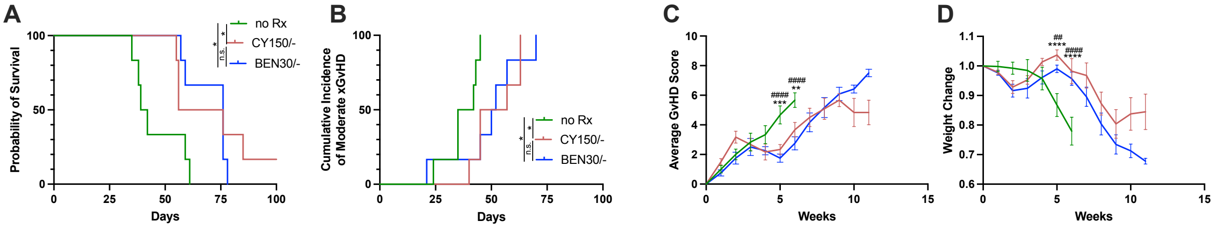

Supplemental Figure 1. *Single day administration of a high dose of CY or BEN modestly improves survival from xGvHD.* NSG mice received no pharmacological agent (no Rx, n=6), a single dose of CY (150mg/kg) on day +3 only (CY150/-, n=6), or a single dose of BEN (30mg/kg) on day +3 only (BEN30/-, n=6) and survival, weight change and xGvHD scores were evaluated. **(A)** Kaplan-Meier survival curve. **(B-C)** GvHD was scored on a scale of 0-10 for activity, fur, posture, skin and weight change. **(B)** A score of 5+ at two consecutive scoring days is considered an incidence of moderate xGvHD, which is illustrated in the graph. **(C)** Average GvHD scores per group over time. **(D)** Average weight change per group over time. **(A-B)** Statistics were done using the Log-rank Mantel-Cox test for survival/incidence curves with p values indicated by the number of asterisks between groups: *<0.05, n.s.= not significant. **(C-D)** Statistics were done with a two-way ANOVA followed by Šídák's multiple comparisons. P values are indicated by the number of symbols between groups noted on graphs, * <0.05, ** <0.01, ***<0.001, ****<0.0001. Asterisks (*) indicate significant differences between no Rx and CY150/-, whereas pound signs (#) indicate differences between no Rx and BEN30/-. **(A-D)** Data is combined from two independent experiments.

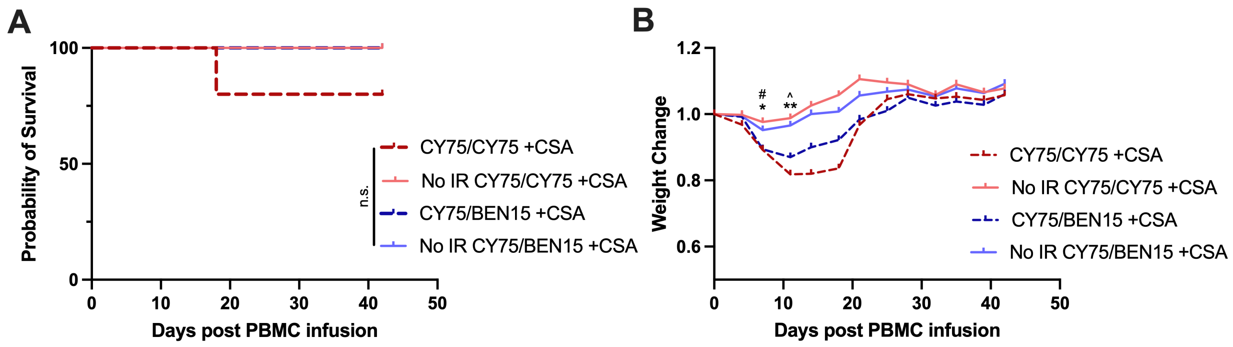

Supplemental Figure 2. *The combination of CY and/or BEN with CSA does not induce toxicity in mice.* Select groups of mice received 150 cGy radiation and were treated with 75mg/kg CY on days +3 and +4 (CY75/CY75 +CSA, n=5), or CY on day +3 and 15mg/kg BEN on day +4 (CY75/BEN15 +CSA, n=5) and then treated with daily cyclosporine A (CSA) for two weeks starting at day +5 and then 3x/week for the remainder of the study. Other groups of mice did not receive radiation but were given the same treatment regimens of CY on days +3 and +4 and CSA thereafter (No IR CY75/CY75 +CSA, n=5) or CY on day +3 and BEN on day +4 and CSA thereafter (No IR CY75/BEN15 +CSA, n=5) and were monitored for survival and weight change. **(A)** Kaplan- Meier survival curve. Statistics were done using the Log-rank Mantle-Cox test for survival curves, n.s.= not significant. **(B)** Change in weight over time. Statistics were assessed using a two-way ANOVA with by Šídák's post-hoc comparisons. P values are indicated by the number of symbols, *<0.05, **<0.01. Asterisks (*) indicate a difference between CY75/CY75 +CSA and No IR CY75/CY75 +CSA. Pound symbols (#) indicate a difference between CY75/BEN15 +CSA and No IR CY75/CY75 +CSA. Arrows (^) indicate a difference between CY75/CY75 +CSA and No IR CY75/BEN15 +CSA.

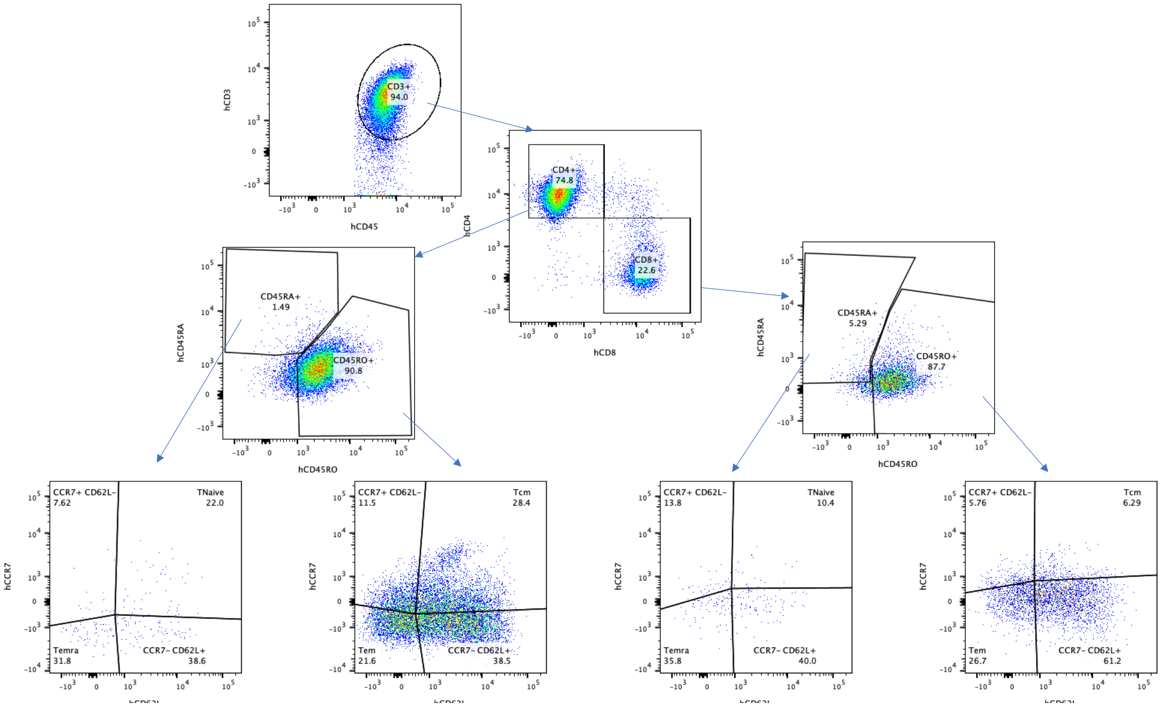

Supplemental Figure 3. *Gating strategy for naïve, effector, effector memory, central memory and undefined T cells populations.* T cells were gated as CD45+CD3+, were differentiated as CD4+ or CD8+ and then further gated into the following subsets: Naïve T cells CD45RA+CCR7+CD62L+, Effector T cells CD45RA+CCR7-CD62L-, Effector Memory T cells CD45RO+CCR7-CD62L-, and Central Memory T cells CD45RO+CCR7+CD62L+ and undefined T cell populations CD45RA+CCR7+CD62L-, CD45RA+CCR7-CD62L+, CD45RO+CCR7+CD62L-, CD45RO+CCR7-CD62L+. Fluorescence minus one (FMO) controls were used to set gates for all experiments.

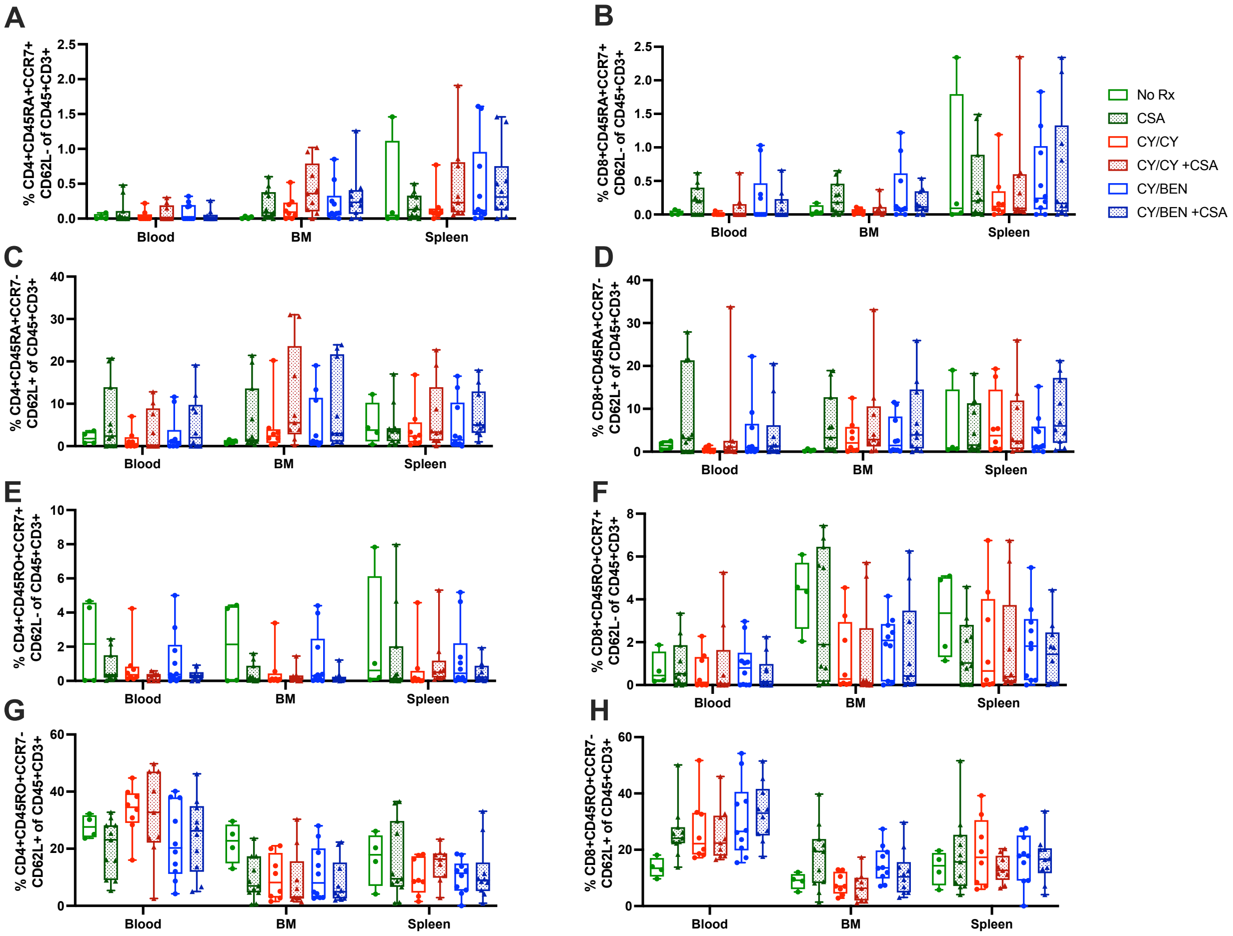

Supplemental Figure 4. *Rates of undefined T cell subsets vary across treatment regimens.* Mice were treated with indicated treatment regimens and blood, bone marrow and spleens were harvested at day 31 for flow cytometric analysis. CD45+CD3+ T cells were gated with indicated markers and currently undefined subpopulations of T cells or perhaps T cells transitioning between phenotypes of naïve, effector, effector memory and central memory subsets were identified as **(A)** CD4+CD45RA+CCR7+CD62L- **(B)** CD8+CD45RA+CCR7+CD62L- **(C)** CD4+CD45RA+CCR7-CD62L+ **(D)** CD8+CD45RA+CCR7-CD62L+ **(E)** CD4+CD45RO+CCR7+CD62L- **(F)** CD8+CD45RO+CCR7+CD62L- **(G)** CD4+CD45RO+CCR7-CD62L+ **(H)** CD8+CD45RO+CCR7-CD62L+. Significant differences were evaluated via a one-way ANOVA followed by Tukey's post-hoc test, with no groups exhibiting statistical differences across treatment groups.

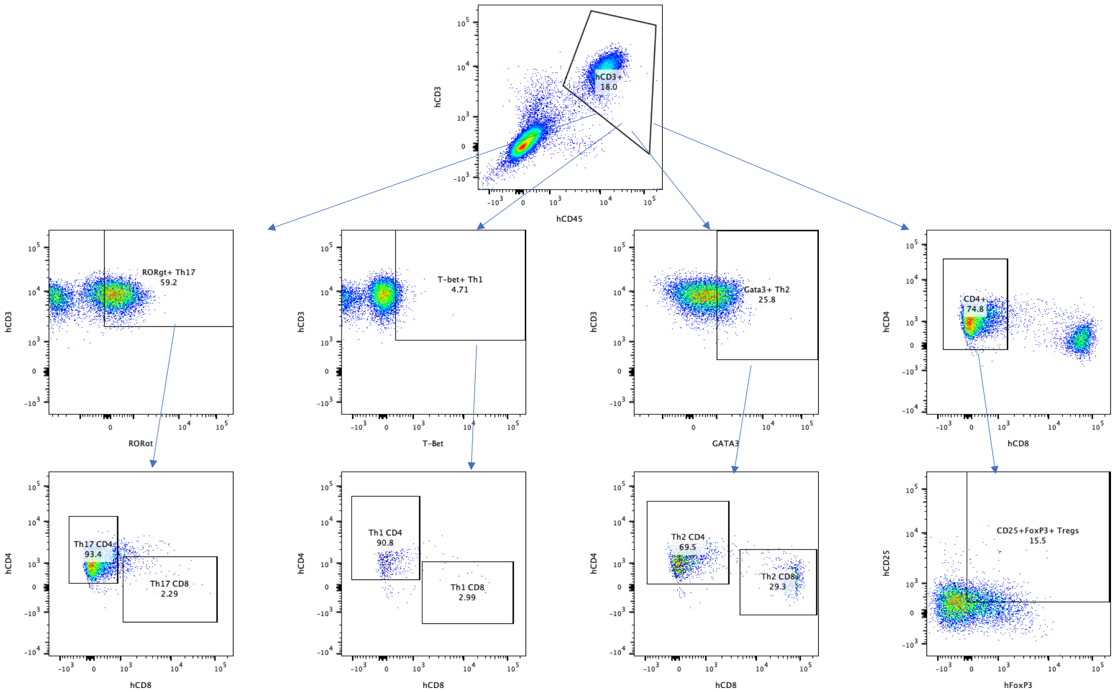

Supplemental Figure 5. *Gating strategy for regulatory T cells and Th1, Th2 and Th17 polarized subsets.* T cells were gated as CD45+CD3+. T regulatory cells were gated as CD4+CD25+FoxP3+. Polarized T cell subsets were gated as T helper 1 cells: T-bet+, T helper 2 cells: GATA3+ and T helper 17 cells: RORγt+ and then sub-gated as CD4+ or CD8+. Fluorescence minus one (FMO) controls were used to set gates for all experiments.

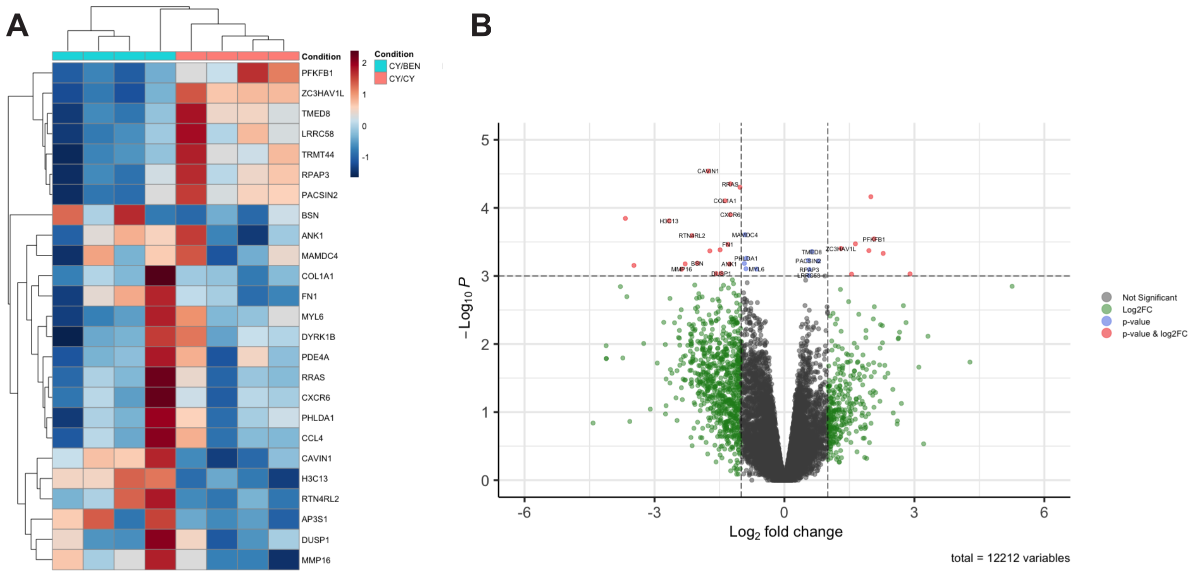

Supplemental Figure 6. *Differential gene expression in CY/CY versus CY/BEN is modestly different at day 31.* Differentially expressed genes from splenic transcriptomes at day 31 following PBMC-infusion. (A) Heat map of top differentially expressed genes between CY/CY and CY/BEN groups with a p value cutoff of *P<0.001* are illustrated. (B) Volcano plot of differentially expressed genes between CY/CY and CY/BEN groups. The horizontal line denotes a P value of *P<0.001*.

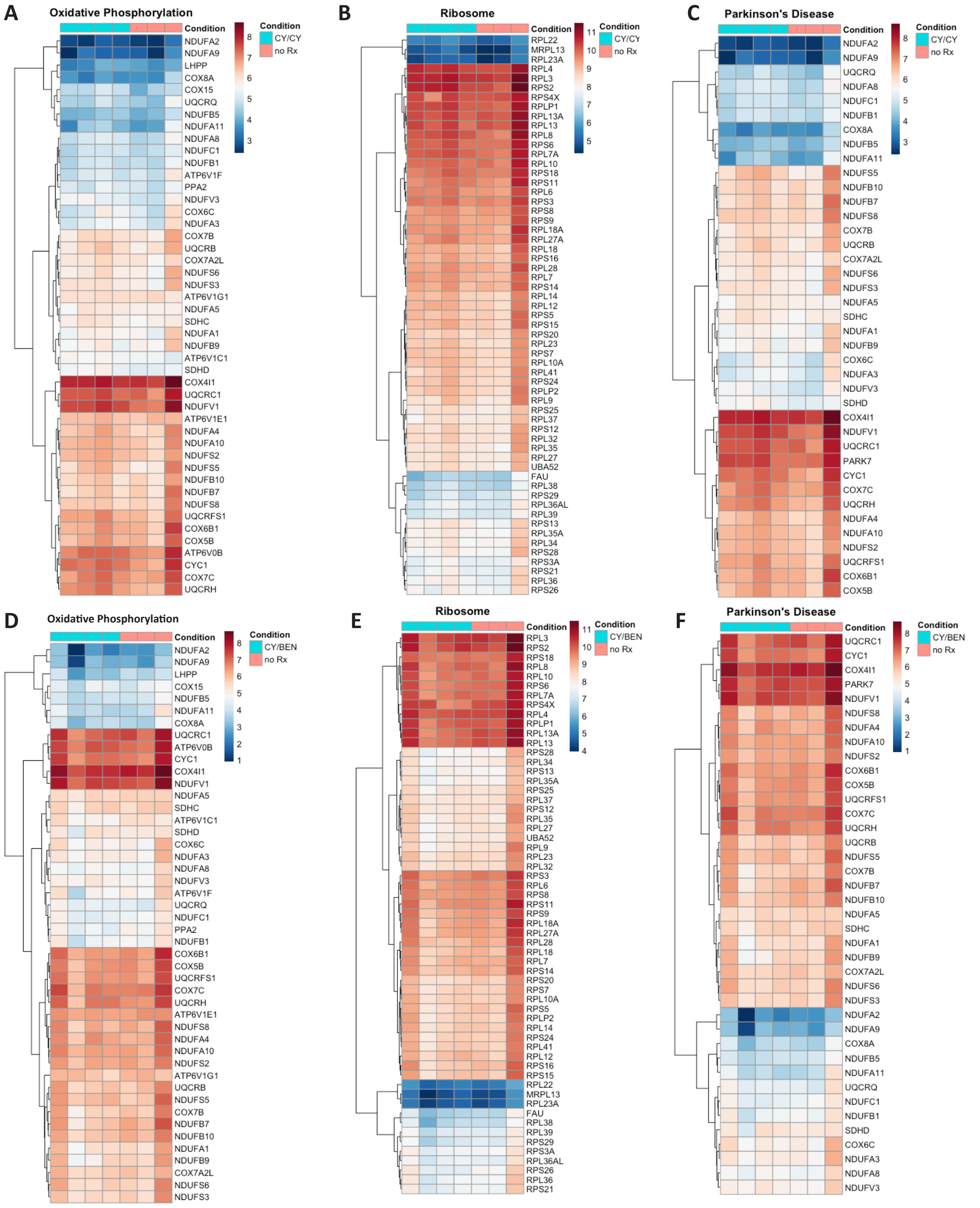

Supplemental Figure 7. *Gene expression differences between no Rx and CY/CY or CY/BEN groups show limited differences between treatment regimens.* Significantly different KEGG terms identified via gene set enrichment analysis from transcriptomes of spleens from **(A-C)** no Rx versus CY/CY or **(D-F)** no Rx versus CY/BEN treated mice at day 31 following PBMC infusion. **(A-F)** GSEA results with a q-value of

q<0.05 are shown as heat maps illustrating differences in GSEA genes associated with specific KEGG pathways generated from log-transformed gene counts using only genes denoted as core enriched for **(A, D)** oxidative phosphorylation, **(B, E)** ribosome, and **(C, F)** Parkinson's disease.
